# Peripersonal space in the front, rear, left and right directions for audio-tactile multisensory integration

**DOI:** 10.1038/s41598-021-90784-5

**Published:** 2021-05-28

**Authors:** Yusuke Matsuda, Maki Sugimoto, Masahiko Inami, Michiteru Kitazaki

**Affiliations:** 1 Department of Computer Science and Engineering, Toyohashi University of Technology, 1-1 Hibarigaoka, Tempaku, Toyohashi, Aichi 441-8580 Japan; 2 Department of Information and Computer Science, Keio University, 3-14-1 Hiyoshi, Kohoku-ku, Yokohama, Kanagawa 223-8522 Japan; 3 Research Center for Advanced Science and Technology, The University of Tokyo, 4-6-1 Komaba, Meguro-ku, Tokyo, 153-8904 Japan

**Keywords:** Human behaviour, Sensory processing

## Abstract

Peripersonal space (PPS) is important for humans to perform body–environment interactions. However, many previous studies only focused on the specific direction of the PPS, such as the front space, despite suggesting that there were PPSs in all directions. We aimed to measure and compare the peri-trunk PPS in four directions (front, rear, left, and right). To measure the PPS, we used a tactile and an audio stimulus because auditory information is available at any time in all directions. We used the approaching and receding task-irrelevant sounds in the experiment. Observers were asked to respond as quickly as possible when a tactile stimulus was applied to a vibrator on their chest. We found that peri-trunk PPS representations exist with an approaching sound, irrespective of the direction.

## Introduction

It is important for human and non-human primates to detect a sensory input that has a potential threat at present or in the near future and to perform body–environment interaction in the space immediately surrounding the body. This space is called the peripersonal space (PPS). The PPS is located between the body surface and the extrapersonal space that is far from the body. Normally, human and non-human primates cannot touch objects placed in the extrapersonal space^1,2^. The PPS clearly has a different role to the extrapersonal space because an event in this area is likely to be involved in human and non-human primates’ actions, such as grasping a doorknob or holding a baby in their hands. Many studies have been conducted on PPS, and all report various characteristic features of PPS.

Multiple studies on neurophysiology in monkeys have reported that there are multisensory neurons in the ventral premotor cortex representing visuo-tactile (visuo-somatic)^3–10^ or audio-tactile (visuo-audio-tactile)^11,12^ information surrounding the monkey’s bodies (PMv; F4^5,6,9,10^), as well as in the ventral intraparietal (VIP) areas^4,12^, parietal areas (7b^3^), and the putamen^7,8^. Graziano & Cooke (2006) have summarized the findings on multisensory neurons in monkeys^13^. These neurons respond only when the information exists within the space immediately surrounding the body parts including the head^4,6,9,11,12^, face^8,10^, and arms (hands)^5,7–9^. Schlack et al. (2005)^12^ showed that macaque VIP neurons respond not only to visual stimuli, but also to auditory stimuli, and that these neurons are bi- or trimodal. Additionally, the PPS mapping varies dynamically in response to external factors^14,15^. Fogassi et al. (1996)^14^ showed that an increase in approaching stimulus velocity produces an expansion in the depth of the receptive field in most neurons in area F4, such that fast-moving stimuli are signaled earlier than slow-moving ones. Iriki et al. (1996)^15^ showed that neuron remapping occurs when trained macaque monkeys attempt to obtain food pellets using a rake. With training, the neuron map shifts to the rake position from the actual hand position to use the rake. However, in the study^15^, the visual stimuli were moved toward or away from the monkey’s hand in a centripetal or centrifugal fashion. Thus, some argue that it is difficult to conclude that the concentration of neuronal responses when the stimulus was near the hand reflects a neuronal response selective for stimuli presented within body-part-centered peripersonal space^16^. Taken together, PPS neurons respond to stimuli within the space immediately surrounding the body or body parts, and the responses change dynamically by external factors.

Behavioral and neurophysiological studies on human participants have reported that various external factors modulate the boundary of the PPS: tool-using^17–20^, wrist weight^21^, gravity or vestibular cues^22,23^, walking^24^, pseudo-walking^25^, grasping^26^, fear of the stimuli^27,28^, and social context^29–31^. For example, Noel et al. (2015)^24^ showed that the space of audio-tactile integration (i.e., the size of the PPS) expanded forward when walking, which it did not when staying in one position. Furthermore, the timescale of the PPS modulation was not only a long-term experience^32^, but also endured from trial-to-trial^33,34^. These facts suggest that, by changing the PPS, humans can adaptively perceive “the space close to themselves”, dynamically in real time, and integrate multisensory information in the space to address not only normal but also emergency situations. Recent studies have shown that the PPS is also effective in virtual environments^35–37^. For example, Serino et al. (2018)^37^ evaluated multisensory tasks in a mixed reality (MR) ecosystem using ecological validity conditions and showed that the results obtained with the MR environment are equivalent to those obtained within real environments. The PPS is related to the field of human factors. Cross-modal looming signals (visual, auditory, and tactile sensations) on human spatial attention of the front and rear near space have been investigated in the field of human factors^38–41^. The cross-modal attention to sounds in the rear (unseen) space is different from that in the front space^41^. These findings contribute to the design of human interfaces by providing appropriate warning signals for safe driving^38^.

Most studies focused on PPS only in the near area in front of the body^27,29,30,32,42^, although multisensory information is available in a variety of directions in the real world^43–46^. Indeed, there are empirical findings that the PPS exists not only in front of the body but also in other directions, as in the study of monkeys^11^ and human studies^47,48^. However, it is not clear whether the PPS is different or identical in all directions (see review^49^). Some studies measured PPS in the rear of the body using audio-tactile or visuo-tactile stimuli and concluded that PPS was asymmetric between the front and rear of the body, and that the PPS in front of the body was larger than that in the rear of the body^50,51^. For example, Kóbor et al. (2006)^51^ showed that non-musicians and pianists performed temporal order judgments between tactile stimuli in their left and right hands when they crossed or uncrossed their hands in front of or behind their body. Both participant groups showed a much reduced crossed-hand interference when they crossed their hands behind their bodies rather than at the front, suggesting that the spatiotemporal representation of the PPS is different between the front and rear spaces. On the other hand, Serino et al. (2015)^52^ investigated PPS representation around the trunk in the front and rear spaces with visuo-tactile stimuli and found that the peri-trunk PPS is symmetric between the front and rear of the body, in that the size of the PPS in front of the body is the same as that in the rear of the body. They also showed that there are three different body-part PPS representations (trunk, hand, and face), and the PPS size was smallest for the hand, larger for the face, and largest for the trunk. Although they did not investigate the hand PPS when the hand was located behind the trunk, hand PPS was modulated according to the relative position to the trunk. Thus, the trunk PPS is larger and more constant than the hand PPS^52^. PPS in the lateral areas (left and right) of the body has been investigated using audio-tactile stimuli^23,53^. Pfeiffer et al. (2018)^23^ showed that the PPS is symmetrical between the left and right spaces with right-handed participants. Hobeika et al. (2018)^53^ focused on handedness and showed that the PPS size of right-handed observers is larger in the left hemispace space than in the right hemispace space, while the PPS size of left-handed observers was the same between the left and right hemispheres. Thus, this anisotropy in the lateral PPS was observed in right-handed observers. The difference between these studies^23,53^ may be due to variations in the degree of handedness of participants and differences in stimuli and setups.

**Figure 1:**
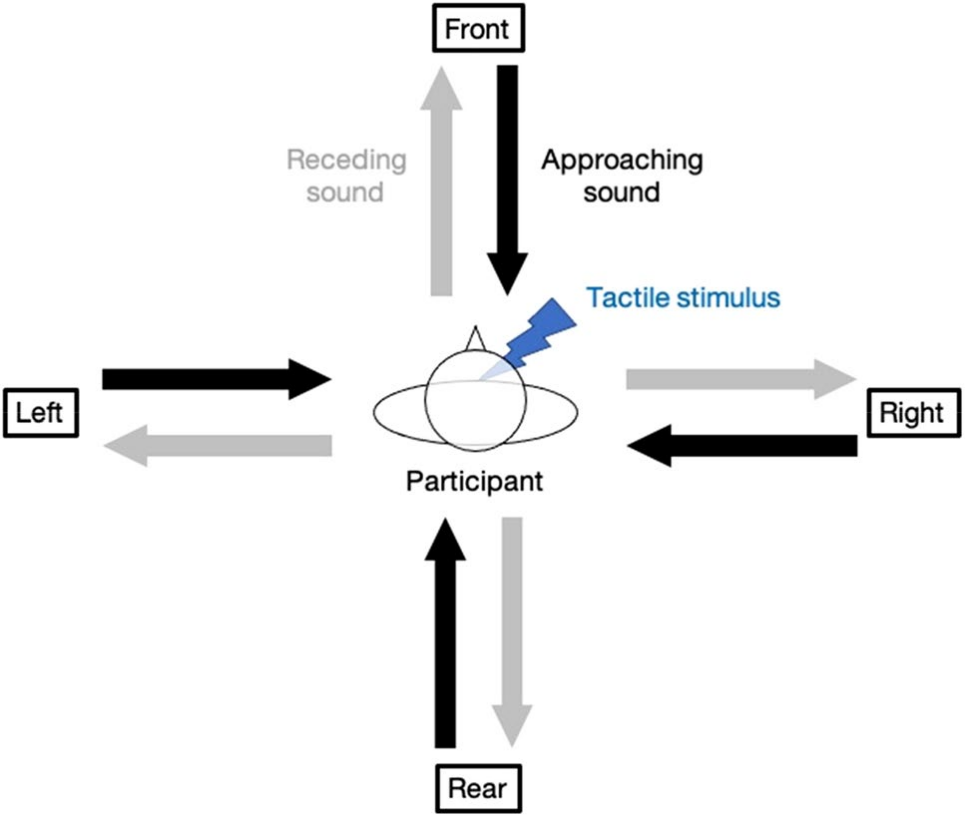
The schematic of the experiment. A virtual audio source was presented. The audio motion was presented toward the participant (approaching sound) or away from the participant (receding sound) in four body-relative directions (front, rear, left, and right). Participants were asked to detect a tactile stimulus presented on the chest as quickly as possible, irrespective of the sound stimulus. Timings of the tactile stimulus and the sound motion were systematically varied.

However, the PPS of all four directions (front, rear, left, and right) has not been compared using the same apparatus. We believe that it is important to investigate the PPS in four directions because humans must detect and perceive auditory and tactile information that occurs in various directions at all times. Thus, we hypothesized that the representation of the peri-trunk PPS for audio-tactile stimuli is a circle centered on the position of the human. To test this hypothesis, we compared the PPS for audio-tactile stimuli in the front of the body with the PPS in the other three directions (rear, left, and right) of the body (Fig. 1). Twenty participants were asked to detect the tactile stimulus on the chest as quickly as possible, irrespective of the auditory stimulus that was either approaching sound or receding sound. The tactile stimulus was presented at seven different temporal delays (T_before_, T_1_, T_2_, T_3_, T_4_, T_5_, and T_after_). The T_1_ – T_5_ tactile stimuli were set at 300, 800, 1500, 2200, and 2700 ms from the sound onset. The T_before_ and T_after_ tactile stimuli were set 700 ms before the sound onset and 700 ms after the sound offset, respectively.

## Results

We analyzed only the RTs in the bimodal and unimodal conditions. We eliminated missing responses (8.74%) from the analysis. We also eliminated the data when the RT was more than 1,300 ms and less than 100 ms (1.03%). We also applied the Grubbs’ test to find and remove outliers before the analysis (1.67%). In total, we excluded 11.4% of the data from the analysis. In catch trials, the false alarm rate was very low (0.96%).

### Effect of body direction on peri-trunk PPS

**Figure 2:**
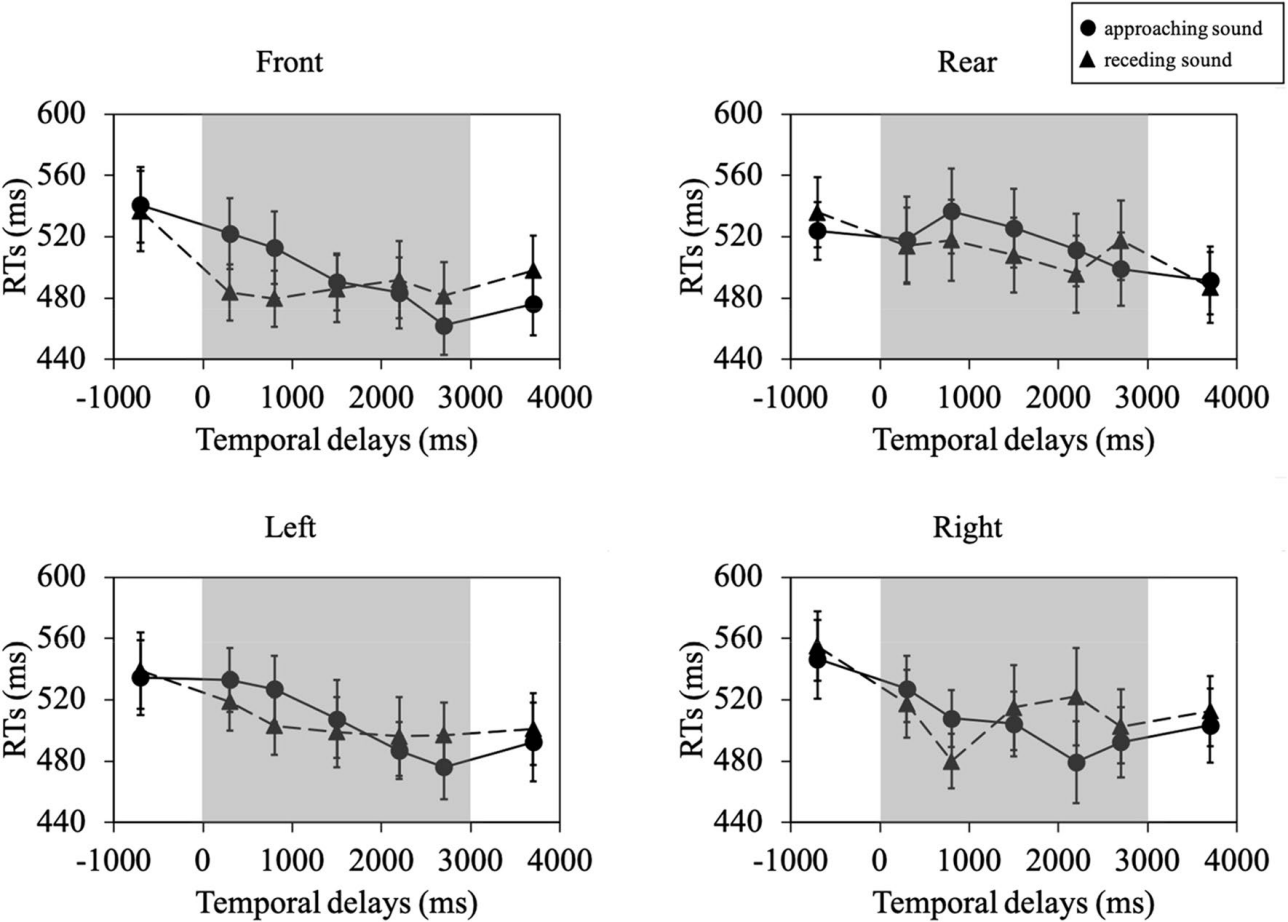
The results of the Experiment RTs (ms) as a function of the temporal delays (ms). Circular and triangular symbols indicate the approaching and receding sounds, respectively. The gray area indicates the timing of the pink-noise emission. Error bars represent standard errors.

We conducted a three-way repeated measures ANOVA on the mean RTs in four body-relative directions (front, rear, left, and right), two audio motions (approaching and receding sounds), and seven temporal delays (T_before_, T_1_, T_2_, T_3_, T_4_, T_5_, and T_after_). The ANOVA revealed a significant main effect of temporal delays (*F* (6, 114) = 4.82, *p* = 0.0002, *η*_*p*_^*2*^ = 0.20, 1-β (power) = 0.99) and a significant interaction of audio motion × temporal delay (*F* (6, 114) = 4.20, *p* = 0.0007, *η*_*p*_^*2*^ = 0.18, 1-β = 0.97, shown in Fig. 2). The other main and interaction effects were not significant (body-relative direction, *F* (3, 57) = 1.11, *p* = 0.35, *η*_*p*_^*2*^ = 0.06, 1-β = 0.31; audio motions, *F* (1, 19) = 0.03, *p* = 0.87, *η*_*p*_^*2*^ = 0.001, 1-β = 0.05; body-relative directions × audio motions, *F* (3, 57) = 1.51, *p* = 0.22, *η*_*p*_^*2*^ = 0.07, 1-β = 0.38; body-relative directions × temporal delays, *F* (18, 342) = 1.45, *p* = 0.10, *η*_*p*_^*2*^ = 0.07, 1-β = 0.90; body-relative directions × audio motions × temporal delays, *F* (18, 342) = 0.81, *p* = 0.68, *η*_*p*_^*2*^ = 0.04, 1-β = 0.60). The results suggest that the direction of sound relative to the body does not affect the PPS representation.

### Differences in peri-trunk PPS between approaching and receding sounds

**Figure 3:**
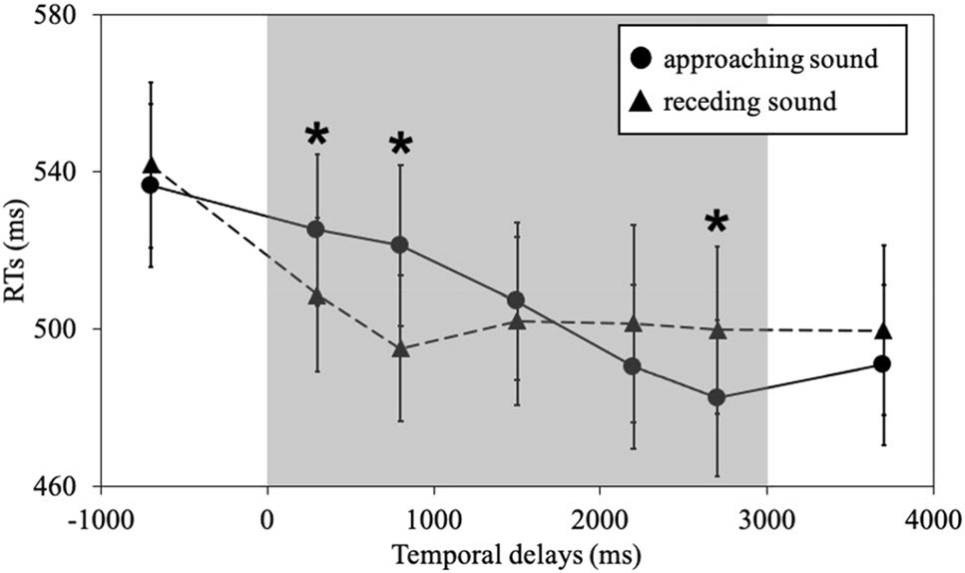
The results of the Experiment RTs (ms) as a function of the temporal delays (ms). The four direction conditions (front, rear, left, and right) were merged. The description of the figure is the same as in Fig. 2. The error bars represent standard errors. Asterisks represent significant differences (*p* < .05) between the RTs for the approaching sound and the receding sounds as a result of multiple comparisons (Shaffer's modified sequentially rejective Bonferroni procedure).

To compare the peri-trunk PPS for the approaching and receding sounds, we conducted an analysis of simple main effects for the significant interaction of audio motions × temporal delays (Fig. 3). The analysis revealed that the mean RTs for the approaching sound were significantly slower than for the receding sound at T_1_ (525 ms vs 509 ms; *F* (1, 19) = 5.08, *p* = 0.036, *η*_*p*_^*2*^ = 0.21, 1-β = 0.57) and T_2_ (521 ms vs 495 ms; *F* (1, 19) = 11.3, *p* = 0.003, *η*_*p*_^*2*^ = 0.37, 1-β = 0.89), while the RTs for the approaching sound were significantly faster than for the receding sound at T_5_ (483 ms vs 500 ms; *F* (1, 19) = 8.60, *p* = 0.009, *η*_*p*_^*2*^ = 0.31, 1-β = 0.79). The results indicated that observers responded to the tactile stimulus faster when the sound position was close to the observers than when the sound position was further away. Thus, the participants could integrate multisensory stimuli (tactile and task-irrelevant audio stimuli) near the body.

### Effect of temporal delays on peri-trunk PPS at approaching or receding sounds

The analysis of simple main effects also revealed that the mean RTs for temporal delays in the approaching and receding sounds were significantly different (approaching: *F* (6, 114) = 6.10, *p* < 0.0001, *η*_*p*_^*2*^ = 0.24, 1-β = 0.99, receding: *F* (6, 114) = 3.45, *p* = 0.004, *η*_*p*_^*2*^ = 0.15, 1-β = 0.93). Post hoc tests (Shaffer's modified sequentially rejective Bonferroni procedure) of the interaction revealed that the mean RTs for the approaching sound at T_5_ (483 ms) were significantly faster than at T_1_ (525 ms) and T_2_ (521 ms) compared to bimodal conditions (T_1_–T_5_). In contrast, there were no significant differences between bimodal conditions for the receding sound. These results indicate that the effect of PPS in the receding sound was never or weaker than that in the approaching sound, which is consistent with the results of previous studies^42,52^.

The analysis also revealed that the RTs for the approaching sound on T_before_ (537 ms) were significantly slower than on T_after_ (491 ms), and RTs for the receding sound at T_before_ (543 ms) were significantly slower than those for T_2_ (495 ms) and T_after_ (500 ms).

### Exploratory analysis on the approaching sound PPS

To investigate the PPS effect of the approaching sound in four directions, we conducted exploratory analyses. First, two-way repeated measures ANOVA was conducted on the mean RTs in four body-relative directions (front, rear, left, and right) and five temporal delays (bimodal condition: T_1_, T_2_, T_3_, T_4_, and T_5_), for only the approaching sound condition. The ANOVA revealed a significant main effect of temporal delays (*F* (4, 76) = 6.79, *p* = 0.0001, *η*_*p*_^*2*^ = 0.26, 1-β = 0.99). The other main and interaction effects were not significant (body-relative directions, *F* (3, 57) = 1.38, *p* = 0.26, *η*_*p*_^*2*^ = 0.07, 1-β = 0.36; body-relative directions × temporal delays, *F* (12, 228) = 0.85, *p* = 0.60, *η*_*p*_^*2*^ = 0.04, 1-β = 0.49). Second, one-way repeated measures ANOVA was conducted on the mean RTs with five temporal delays (bimodal condition: T_1_, T_2_, T_3_, T_4_, and T_5_) separately for each of the four directions (front, rear, left, and right), for only the approaching sound condition. The ANOVA revealed a significant main effect of temporal delays in front (*F* (4, 76) = 5.12, *p* = 0.001, *η*_*p*_^*2*^ = 0.21, 1-β = 0.95) and left space (*F* (4, 76) = 6.54, *p* = 0.0001, *η*_*p*_^*2*^ = 0.26, 1-β = 0.99), while the main effects of temporal delays were not significant in the rear (*F* (4, 76) = 1.12, *p* = 0.35, *η*_*p*_^*2*^ = 0.06, 1-β = 0.36) or right (*F* (4, 76) = 1.78, *p* = 0.14, *η*_*p*_^*2*^ = 0.09, 1-β = 0.54) directions. These results indicate that the PPS might be affected by the direction, and clearer in the front and left directions than in the rear and right directions. However, we did not find a significant interaction of the direction and the time delay with two-way repeated measures ANOVA, as mentioned above.

**Figure 4:**
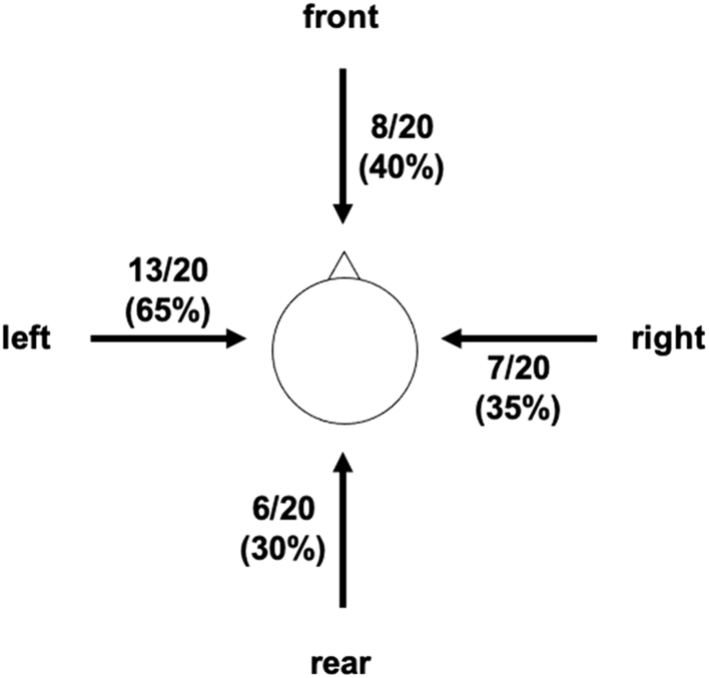
The number of observers (N) of all 20 participants (N/20) and its percentage (%) with R^2^ (coefficient of determination for the sigmoidal function) higher than 0.5 for each body-relative direction for the approaching sound condition. The results of Table 1 and these results, taken together, imply that the PPS in the front and left directions might be stronger while the PPS of the rear might be weaker.

To investigate the PPS in each direction in detail, two further analyses were conducted. First, the sigmoidal function was fitted to RTs against temporal delays for the average data across observers. The function is described by the following equation:

$$y\left(x\right)=\frac{{y}_{min}+{y}_{max}\cdot {e}^{\left(x-{x}_{c}\right)/b}}{1+{e}^{\left(x-{x}_{c}\right)/b}}$$

where *x*_*c*_ represents the value of the abscissa at the central point of the function and b represents the slope of the function at the central point^29,42^. The slope of the sigmoidal function is large when the absolute value of *b* is small. Table 1 shows the slopes of the sigmoid function for each condition. The absolute *b* values in the front and left conditions were smaller than those in the rear and right conditions. Namely, the slopes of the sigmoidal function in the front and left conditions were larger than those in the rear and right conditions. Second, the sigmoidal function was fitted for each observer, and we calculated the coefficient of determination (R^2^) for the sigmoidal function, based on the analysis of Serino et al. (2018)^37^. Figure 4 shows the number of observers with R^2^ higher than 0.50. We found that the number of observers showed that the clear PPS (higher R^2^) was less in the rear direction than in the other three directions. Taken together, the results of the present study suggest that the PPS of the front and left directions might be stronger while the PPS of the rear might be weaker, although the analysis was exploratory.

**Table 1:** The data of slopes of the sigmoidal function (*b*) in each direction.

Directions	Front	Rear	Left	Right
*b*	− 492.1	− 510.6	− 362.0	− 548.8

## Discussion

Tactile detection was facilitated by task-irrelevant approaching sounds in the near body space rather than by receding sounds in all four directions (front, rear, left, and right). We did not find any significant differences in the directions of the PPS representation (front, rear, left, and right). These results suggest that humans integrate multisensory information in near-circular peri-trunk PPS around the body, and that integration occurs with approaching sounds.

Physiological and behavioral studies on humans and non-human primates have shown that approaching stimuli (for example, visual and auditory stimuli) in near space increase sensitivity to different sensory modalities (for example, tactile stimuli)^29,30,43,54,55^. The cortical network that processes approaching visual stimuli in the PPS to predict the consequences of these stimuli has been identified^56,57^. These findings are supported by an ecological perspective; the approaching stimulus contributes to detecting a danger or a predator approaching humans or animals as an information alert and predicting its timing^55–57^. Our findings are also consistent with these studies. Tactile detection was facilitated by approaching sounds in the near body space, but the effects of PPS in the receding sound were weaker than those in the approaching sound. This suggests that a receding event, even though happening in the near space around humans, would not facilitate the detection of the event in different sensory modalities. This is because the event is receding, and hence is not considered to involve humans in the near future.

One might argue that the sound provided temporal alerting information even though the sound was designed as a task-irrelevant stimulus. Particularly, the approaching stimulus can be an alert of an event approaching and contribute to predicting its timing, as discussed in the previous paragraph. However, we instructed the participants to neglect the sound and respond only to the tactile stimulus so that the sound was defined as a task-irrelevant stimulus. Moreover, the tactile stimulus was not presented in 30% of the trials and was presented before the sound in the T_before_ condition. For the sound to be used as a temporal timing indicator, the RTs of the T_before_ condition must have been longer than the bimodal conditions (T_1_–T_5_). We did not find significant differences between bimodal and unimodal conditions, except between T_before_ and T_2_ for the receding sound. Thus, it is unlikely that the sound contributed to the prediction of the timing of the tactile stimulus, particularly in the approaching sound condition.

We placed the vibrator on the chest (upper front part of the body) in all directions (front, rear, left, and right). Thus, one might expect the facilitation of tactile detection to occur only in the front condition; however, we found facilitation in all directions. It is suggested that the PPS representation is nearly circular around the trunk of the body. A previous study indicated that the peri-trunk PPS was distributed in a relatively large space without a difference between the front and rear of the trunk^52^. Our results support this hypothesis.

By using both approaching and receding stimuli, one may argue that tactile stimulus expectancy could be used to interpret the results of the study. Indeed, some previous studies have indicated that expectancy occurred under the experimental method we used (called expectancy effect^58^). This could explain why T_after_ was faster than T_before_. If the expectancy effect modulates the tactile detection in bimodal conditions, it conflicts with the effect of the PPS representation with receding sounds. In our results, the effect of the PPS with receding sounds was weaker than that with approaching sounds. Thus, our results would be caused not only by the PPS representation but also the expectancy effect. However, the false alarm rate in the catch trials (the ratio of participants’ responses without tactile stimulus) was very low in this study. Therefore, the expectancy effect was almost suppressed in the results of our study. Nevertheless, the advantage of approaching sounds could be explained by the ecological validity of the approaching sound. The looming or approaching stimuli are more likely to be relevant to, or ecologically important for humans and non-human primates than the descending or receding stimuli, and an attentional bias toward approaching stimuli is stronger than the receding stimuli (e.g.,^9,14,59–62^). This is an open question that needs further investigation.

Specific cortical processes for encoding near and far spaces have been investigated using humans and non-human primates, and compared between humans and non-human primates or between species^57,63,64^. Guipponi et al. (2013)^64^ have shown that the VIP area of macaques processes moving objects around and towards the face regardless of the sensory modality and argued that there is a strong homology between the VIP organizations of macaques and humans. Although there is a difference in the body part studied between their study and our study (face vs. trunk), we showed that humans also responded sensitively in the near space to the approaching stimulus. However, it is unclear whether the cortical network for humans is the same as that for monkeys, as this study did not take a neurological approach. In fact, it has been shown that there may be species differences between monkeys and humans^63^. This is one of the research topics that we should pay attention to in the future.

Finally, this study has some limitations. We focused on the relatively near space (10–110 cm) in four directions at chest height (sternum level) to represent the peripersonal space. We thus measured only the peri-trunk PPS. However, various body parts (hands, arms, and head; see review paper in detail^65^) also modulate the boundary of the PPS. Thus, our results cannot be applied to the PPS of other body parts, and this should be therefore investigated in a future study. A recent study has questioned the existence of audio-tactile interactions in the peripersonal space^66^. Thus, the conditions of emerging effects of peripersonal space are limited by various factors and stimulus parameters; therefore, we must be careful to compare different studies, and systematic pre-registered studies are required in the future. Another limitation was that a blocked design was used in the current study. The body-relative direction of the sound was constant at either the front, rear, left, or right in each block. It should be investigated with a trial-by-trial design in future studies because some cognitive phenomena do not occur in a blocked design. For example, attentional cueing effects only occur when stimulus location varies on a trial-by-trial basis^67^. In the future, it would be useful to investigate the PPS representation under the condition that participants cannot predict where the sound located. The tactile stimulus was presented to the front of the chest in all conditions to control sensitivity of touch. However, one may argue that the tactile stimulus should be presented at the trunk in the same direction as the auditory stimulus (e.g., vibration to the right side of the trunk when the sound moves to the right). All directional PPS can be investigated in this manner if we can equalize the strength of tactile stimuli in participants’ sensitivity at various surfaces of the trunk. We have investigated PPS representations in four directions (front, rear, left, right), but not in the up and down directions. PPS representations in three-dimensional space should be investigated in future studies because audio information is available in all directions.

## Methods

### Participants

Twenty observers (all male) aged 19–26 (mean 22.3, SD 1.9) years with a healthy auditory and tactile perception participated in the experiment. All volunteers provided written informed consent before the experiment. This study was approved by the Ethical Committee on Human-Subject Studies of Toyohashi University of Technology, and all methods were carried out in accordance with relevant guidelines and regulations. The sample size was based on previous studies in which a similar measurement of PPS was used^42^. This sample size corresponds to an effect size (f) of 0.12 (*η*_*p*_^*2*^ = 0.015), an alpha of 0.05, and power of 0.95 using G*Power 3.1^68,69^.

### Stimuli

We adopted the audio-tactile task to measure the PPS representation developed by Canzoneri et al. (2012)^42^; participants responded as quickly as possible when approaching or receding sounds resulted in a tactile stimulus on their chest. We used Unity 5.6.1f1 in a computer to control the experimental sequence.

#### Audio stimuli

**Figure 5:**
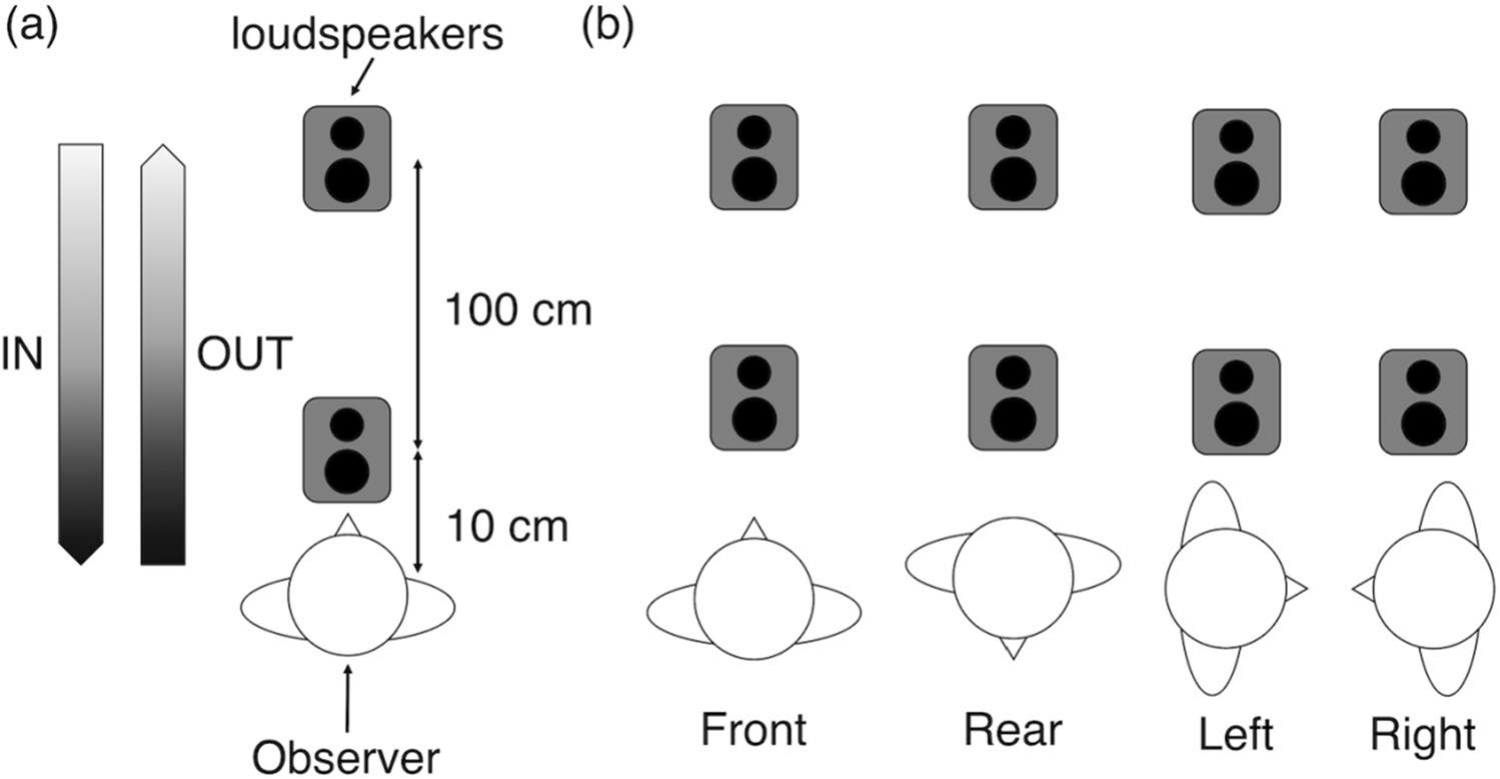
Experimental design. (**a**) Layout of the loudspeakers and observers. (**b**) Direction to the loudspeakers.

Audio stimuli were created by a pink noise (44.1 kHz) that moved toward the observer (approaching sound) and a sound that moved away from the observer (receding sound) for 3,000 ms. We used two loudspeakers (JBL, Control 1 Pro): one was placed close to the participant’s body at a distance of 10 cm, while the other was placed at a distance of 110 cm (Fig. 5a). The loudspeakers were placed at chest height (sternum level). The two loudspeakers were used to present the moving sound. The sound pressure level increased and decreased exponentially in the range of 55–70 dBA for the approaching sound and decreased for the receding sound, respectively. The pink noise moved in a linear uniform motion at 33.3 cm/s between 10 and 110 cm for 3000 ms.

#### Tactile stimuli

In 70% of the trials, the tactile stimulus (a linear vibration actuator Nidec LD14-002) was controlled by the Arduino UNO and presented on the observer’s chest for 24 ms. To eliminate the effect of sensitivity differences for the tactile stimulus (chest, back, and shoulder), the actuator was always placed on the participant’s chest with surgical tape.

There were seven different temporal delays (T_before_, T_1_, T_2_, T_3_, T_4_, T_5_, and T_after_). The T_1_–T_5_ tactile stimuli were set at 300, 800, 1500, 2200, and 2700 ms from the sound onset. The T_before_ and T_after_ tactile stimuli were set 700 ms before the sound onset and 700 ms after the sound offset, respectively. We confirmed that the observers could perceive the vibration before the experiment.

### Conditions

To measure the PPS representations separately in four directions, observers sat on a stool facing a different direction (front, rear, left, or right) to the loudspeakers in each block (Fig. 5b). Thus, the experiment consisted of four blocks. One block was composed of three different trials: bimodal, unimodal, and catch trials. Half (50%) of all the trials were bimodal trials, in which the tactile stimulus was presented with an audio stimulus (2 audio motions (approaching/ receding) × 5 temporal delays (T_1_-T_5_) × 10 repetitions = 100 trials); 20% of all trials were unimodal trials, in which the tactile stimulus was presented without audio stimulus (2 audio motions × 2 temporal delays (T_before_/T_after_) × 10 repetitions = 40 trials); 30% of all trials were catch trials (to avoid easy prediction of timing of given tactile stimulus, see Discussion in detail), in which the tactile stimulus was not presented (60 trials). A block was composed of 200 trials (100 bimodal trials + 40 unimodal trials + 60 catch trials). Thus, observers performed 800 trials (200 trials/block × 4 different body-relative direction) in total.

### Procedure

Participants sat on a stool and blindfolded during the experiment. They were instructed to detect the tactile stimulus on the chest, irrespective of the auditory stimulus, and respond as quickly as possible. They were also instructed not to respond without tactile stimulus (catch trials).

There were breaks after every 50 trials and between blocks. It took approximately 33 min to pass through each block. It took approximately 2.5 h to complete all four blocks. The order of the blocks was counterbalanced among participants, and the order of the trials in each block was randomized.

## Data Availability

The data that support the findings of this study are available from the corresponding author upon reasonable request.
